# Effect of Neprilysin Inhibition for Ischemic Mitral Regurgitation after Myocardial Injury

**DOI:** 10.3390/ijms22168598

**Published:** 2021-08-10

**Authors:** Sahmin Lee, Hyo-Sook Hwang, Naaleum Song, Geun-Hyung Kang, Kyoung-Hee Choi, Eunhye Ji, Jong-Min Song, Duk-Hyun Kang

**Affiliations:** 1 Division of Cardiology, Heart Institute, Asan Medical Center, University of Ulsan College of Medicine, Seoul 05505, Korea; bubbleplusster@gmail.com (H.-S.H.); aleum31@naver.com (N.S.); rkdrmsgud@nate.com (G.-H.K.); brightbrave@hanmail.net (K.-H.C.); jieunhye7@gmail.com (E.J.); jmsong@amc.seoul.kr (J.-M.S.); dhkang@amc.seoul.kr (D.-H.K.); 2 Department of Medical Science, Asan Medical Institute of Convergence Science and Technology, Asan Medical Center, University of Ulsan College of Medicine, Seoul 05505, Korea

**Keywords:** neprilysin, angiotensin receptor antagonists, mitral valve insufficiency, heart failure, heart ventricles

## Abstract

Angiotensin receptor neprilysin inhibitor (ARNI) treatment reduces functional mitral regurgitation (MR) to a greater extent than angiotensin receptor blocker (ARB) treatment alone, but the mechanism is unclear. We evaluated the mechanisms of how ARNI has an effect on functional MR. After inducing functional MR by left circumflex coronary artery occlusion, male Sprague Dawley rats (*n* = 31) were randomly assigned to receive the ARNI LCZ696, the ARB valsartan, or corn oil only (MR control). Excised mitral leaflets and left ventricle (LV) were analyzed, and valvular endothelial cells were evaluated focusing on molecular changes. LCZ696 significantly attenuated LV dilatation after 6 weeks when compared with the control group (LV end-diastolic volume, 461.3 ± 13.8 µL versus 525.1 ± 23.6 µL; *p* < 0.05), while valsartan did not (471.2 ± 8.9 µL; *p* > 0.05 to control). Histopathological analysis of mitral leaflets showed that LCZ696 strongly reduced fibrotic thickness compared to the control group (28.2 ± 2.7 µm vs. 48.8 ± 7.5 µm; *p* < 0.05). Transforming growth factor-β and downstream phosphorylated extracellular-signal regulated kinase were also significantly lower in the LCZ696 group. Consequently, excessive endothelial-to-mesenchymal transition (EndoMT) was mitigated in the LCZ696 group compared to the control group and leaflet area was higher (11%) in the LCZ696 group than in the valsartan group. Finally, the MR extent was significantly lower in the LCZ696 group and functional improvement was observed. In conclusion, neprilysin inhibitor has positive effects on LV reverse remodeling and also attenuates fibrosis in MV leaflets and restores adaptive growth by directly modulating EndoMT.

## 1. Introduction

After myocardial infarction (MI), tethering and fibrosis of mitral leaflets stimulate functional mitral regurgitation (MR), resulting in high morbidity of heart failure (HF) and cardiac mortality [1,2,3,4,5,6,7,8]. As secondary functional MR usually develops as a result of left ventricular (LV) dysfunction [1,6], medications for HF such as beta blockers, angiotensin-converting-enzyme (ACE) inhibitors, and angiotensin receptor blockers (ARBs) are the mainstay of medical therapy for functional MR [9,10]. However, the pharmacological treatment has not been found to be sufficient for reducing ischemic MR or reversing the adverse LV remodeling [5,11,12].

Post-MI changes in the mitral valve (MV) are associated not only with LV remodeling, but also with an excessive endothelial-to-mesenchymal transition (EndoMT) by transforming growth factor-β (TGF-β) overexpression [13,14]. Fibrotic remodeling and thickened leaflet of MV make leaflet area insufficient and cause inadequate adaptation, which finally results in coaptation failure of mitral leaflets and facilitates functional MR [15,16,17,18,19,20]. Therefore, inhibition of EndoMT by blocking TGF-β expression or its downstream signaling may offer a strategy to prevent inadequate adaptations and effectively mitigate functional MR.

Reducing functional MR is highly beneficial to the clinical outcomes of patients with MI or HF. A recent randomized trial found that reduction of functional MR by transcatheter MV repair resulted in a lower rate of hospitalization for HF and lower mortality in patients with HF and significant secondary MR [21]. Furthermore, we recently performed a double-blind, randomized clinical trial, which demonstrated that angiotensin receptor-neprilysin inhibitor (ARNI) treatment effectively reduced functional MR more than ARB alone in patients with LV dysfunction [22]. Although ARNI therapy has shown potential to promote reverse remodeling of LV in this clinical trial, little is known about the mechanism responsible for the beneficial action of neprilysin inhibition on functional MR.

ARNI is a combination drug of sacubitril, a neprilysin inhibitor, and valsartan, an ARB [23]. In the PARADIGM-HF trial [24], ARNI treatment was found to have substantial benefits in reducing all-cause mortality and hospitalization among HF patients with reduced ejection fraction when compared to enalapril, an ACE inhibitor. In addition to the effect as an ARB, the mechanism of beneficial action of neprilysin inhibition includes enhancement of endogenous natriuretic peptide, which facilitates sodium excretion and has vasodilating effects [25,26], and inhibition of cardiac fibrosis and hypertrophy [27]. Natriuretic peptides are hormones produced from heart or vascular endothelium in response to preload or afterload, and fluid retention through specific receptors [28].

The aim of the present study is to examine the hypothesis that treatment of neprilysin inhibitor attenuates functional MR after MI not only by facilitating LV reverse remodeling, but also by mitigating inadequate leaflet adaptation by suppressing EndoMT. In this study, functional MR and LV remodeling after MI were quantified in vivo in Sprague Dawley (SD) rats by using animal echocardiography and cardiac magnetic resonance imaging (MRI). Additionally, primary cultured human valvular endothelial cells were utilized to evaluate the beneficial action mechanism of neprilysin inhibition with a focus on molecular changes in vitro.

## 2. Results

### 2.1. Left Ventricular Remodeling after Myocardial Infarction

**Figure 1:**
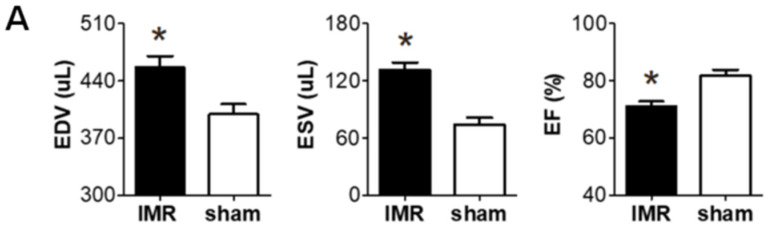

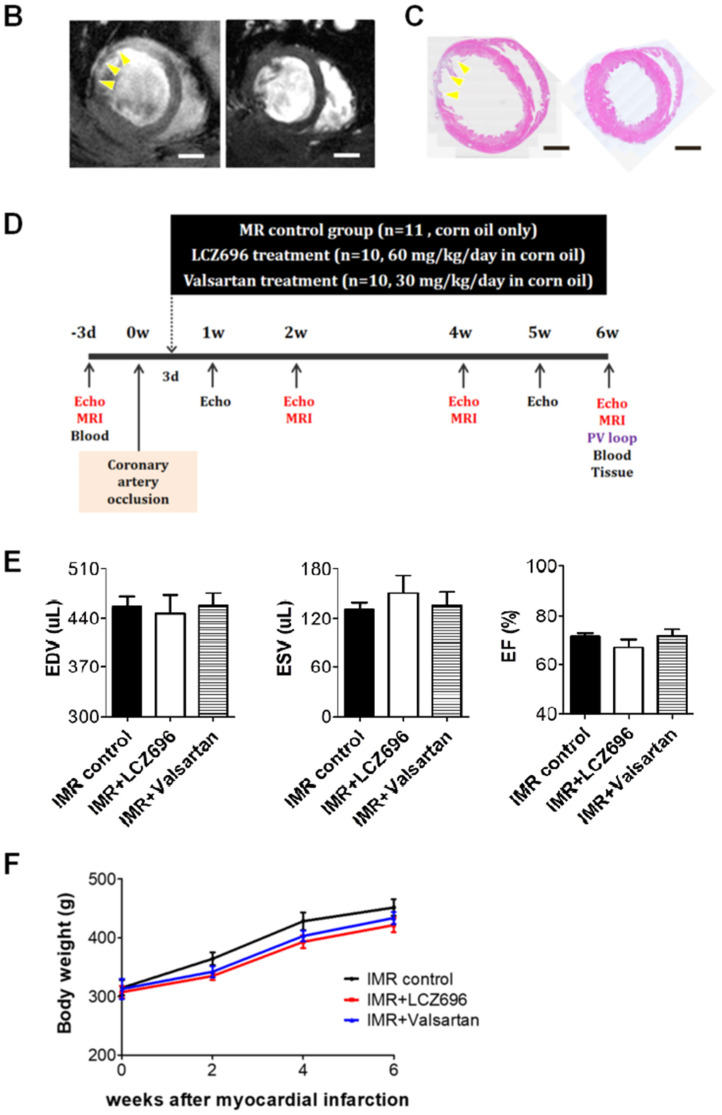
(**A**) Two weeks after left circumflex coronary artery occlusion was performed to induce myocardial infarction and ischemic mitral regurgitation (IMR), left ventricle size and function were evaluated by magnetic resonance imaging (MRI). (**B**,**C**) Representative images of cardiac MRI and immunohistochemical staining of hearts from the IMR model group (left) and sham control group (right). Arrows indicate infarcted area and myocardial fibrosis. Scale bar = 3 mm. (**D**) Schematic illustration of animal experiment protocol. Rats were randomly assigned to LCZ696 treatment (sacubitril/valsartan, 60 mg/kg/day in corn oil, *n* = 10), valsartan treatment (30 mg/kg/d in corn oil, *n* = 10), or corn oil only (MR control group; *n* = 11) group. (**E**) Comparison of parameters at 2 weeks after myocardial infarction among the three groups. (**F**) Comparison of serial changes in body weight among the three groups. EDV indicates end-diastolic volume; ESV, end-systolic volume; EF, ejection fraction; MRI, magnetic resonance imaging; IMR, ischemic mitral regurgitation. * *p* < 0.05 for differences from the sham group.

Two weeks after occluding the left circumflex coronary artery in SD rats, significant post-MI LV dilatation was confirmed (Figure 1A) along with wall motion abnormality and ischemic fibrosis in posterolateral wall of LV (Figure 1B,C). The schematic of the study protocol is illustrated in Figure 1D. There was no significance difference in LV dilation and systolic function between the three groups at 2 weeks (Figure 1E). Six weeks after MI and randomization, all of 31 experimental rats survived well and there was no significant difference in serial changes of body weight between the three groups; LCZ696 treatment, valsartan treatment, and MR control group (Figure 1F).

### 2.2. Neprilysin Inhibitor Facilitates Left Ventricular Reverse Remodeling

**Figure 2:**
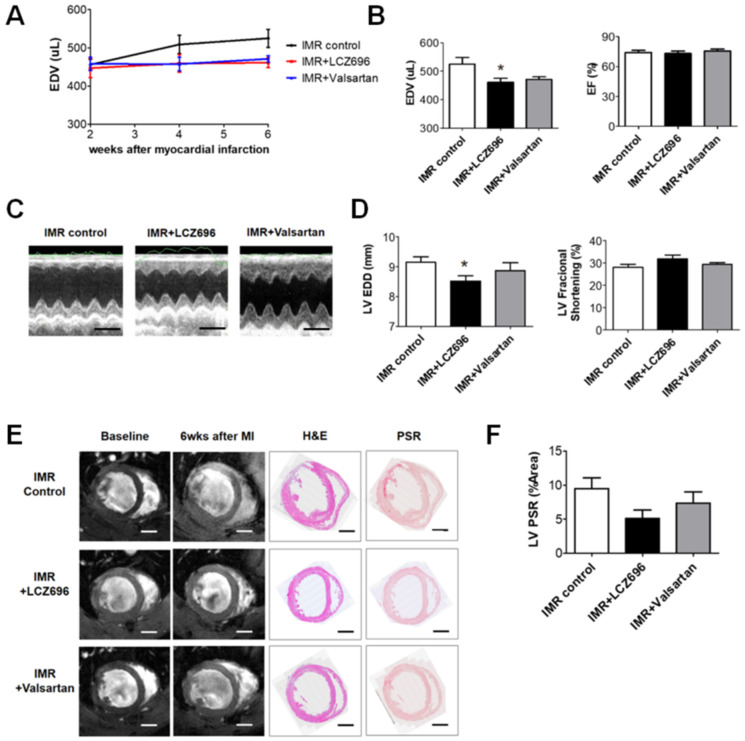

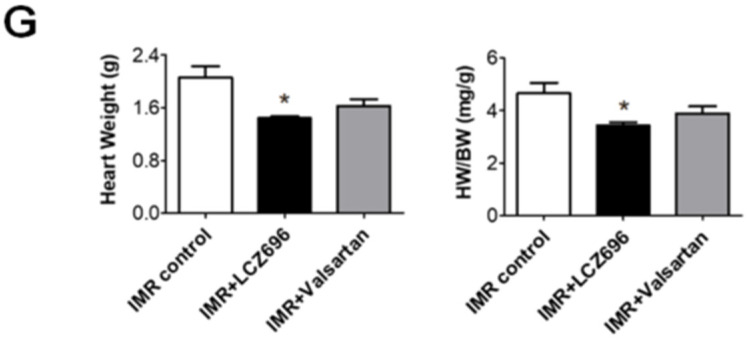
(**A**) Comparison of serial changes in end-diastolic volume (EDV) of the left ventricle until 6 weeks after myocardial infarction (MI) among the three groups. (**B**) Quantitative graphs of cardiac magnetic resonance imaging (MRI) measurements, which showed that EDV was significantly lower in the LCZ696 group than in the control group. There were no significant differences between the valsartan group and the control group. (**C**) Comparison of representative M-mode images acquired by echocardiogram at 6 weeks after MI from the three groups. Scale bar = 5 mm. (**D**) Quantitative graphs of echocardiographic measurements, which showed that end-diastolic dimensions were also significantly lower in the LCZ696 group than in the control group. (**E**) Representative images of cardiac MRI at baseline and 6 weeks after MI and immunohistochemistry at 6 weeks from the three groups. Scale bar = 3 mm. (**F**) A quantitative graph, which shows that the extent of myocardial fibrosis was smaller in the LCZ696 group than in the control group. LV PSR means overall fibrotic area of LV myocardium regardless of infarct size. (**G**) The LCZ696 group also had significantly lower heart weight than the control group, while valsartan did not. EDV indicates end-diastolic volume; IMR, ischemic mitral regurgitation; EF, ejection fraction; LV, left ventricle; EDD, end-diastolic dimension; wk, week; MI, myocardial infarction; H&E, Hematoxylin and eosin stain; PSR, picrosirius red stain; HW, heart weight; BW, body weight. * *p* < 0.05 for differences from the control group.

LCZ696 significantly attenuated post-MI LV dilatation after 6 weeks when compared with the control group (LV EDV, 461.3 ± 13.8 µL versus 525.1 ± 23.6 µL; *p* < 0.05), which was assessed by means of cardiac MRI, while valsartan did not (LV EDV, 471.2 ± 8.9 µL; *p* > 0.05 to control) (Figure 2A,B). Echocardiography also showed significant reduction of LV end-diastolic dimension (EDD) in LCZ696 group (8.5 ± 0.2 mm in the LCZ696 group versus 9.1 ± 0.2 mm in the control group; *p* < 0.05) (Figure 2C,D). LCZ696 treatment decreased post-MI fibrosis in the LV myocardium (Figure 2E,F) as well as gross heart weight compared to the control group (1.4 ± 0.03 g versus 2.1 ± 0.17 g; *p* < 0.05), whereas valsartan did not (1.6 ± 0.10 g; *p* > 0.05) (Figure 2G).

### 2.3. Neprilysin Inhibitor Suppresses Excessive Endothelial-to-Mesenchymal Transition which Mitigates Inadequate Leaflet Adaptation

**Figure 3:**
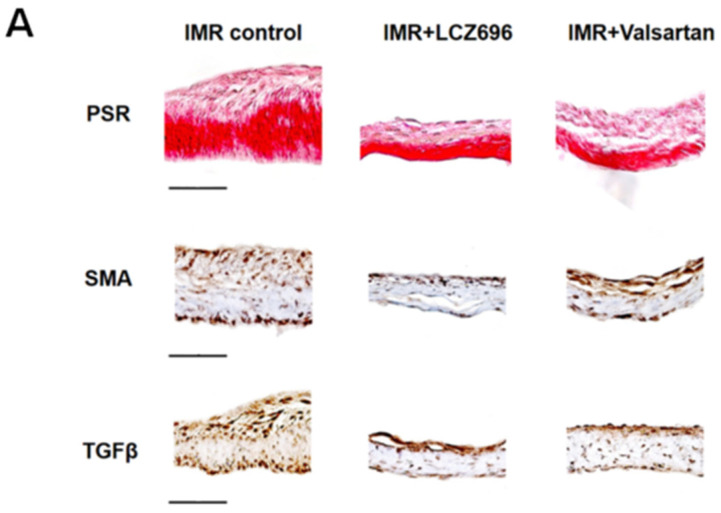

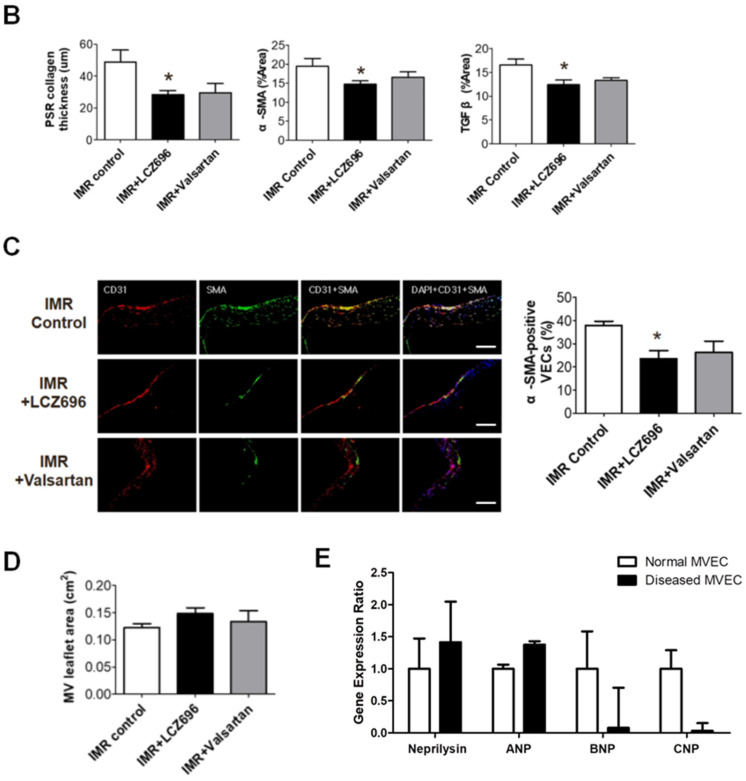
(**A**) Comparison of representative light microscopy image from immune-histochemical staining of the mitral leaflets among the three groups. Scale bar = 50 μm. PSR staining demonstrates marked fibrotic thickening of the mitral leaflets in the IMR control group. This thickening is associated with an increase in excessive endothelial-to-mesenchymal transition represented by α-SMA and TGF-β immunostains. (**B**) Quantification of the PSR collagen thickness (μm), the α-SMA- and the TGF-β-positive areas (% of the mitral leaflet). (**C**) Representative immunofluorescence staining images of CD31 and α-SMA in the mitral leaflets from the three groups. Scale bar = 50 μm. Nuclei were stained with DAPI (blue). Merged images are shown and quantitative graph indicates the proportion of α-SMA(+) CD31(+) cells of total CD31(+) endothelial cells. (**D**) Comparison of mitral leaflet area among the three groups of animal experiments. (**E**) Gene expression levels of neprilysin and natriuretic peptides from hMVECs. IMR indicates ischemic mitral regurgitation; PSR, picrosirius red stain; α-SMA, α-smooth muscle actin; TGF-β, transforming growth factor-β; CD31, cluster of differentiation 31; DAPI, 4′,6-diamidino-2-phenylindole; VEC, valvular endothelial cell; hMVEC, human mitral valvular endothelial cell; ANP, A-type natriuretic peptide; BNP, B-type natriuretic peptide; CNP, C-type natriuretic peptide. * *p* < 0.05 for differences from the control group.

Histopathological analysis of mitral leaflets showed that fibrosis was markedly less prominent in the LCZ696 group than in the MR control group (Figure 3A). The fibrotic thickening of mitral leaflet is associated with an increase in excessive EndoMT, which was represented by α-SMA and TGF-β immunostains. LCZ696 strongly reduced leaflet thickness as well as, α-SMA and TGF-β expression on the mitral leaflets (Figure 3A,B). Using immunofluorescence staining, we also found that LCZ696 significantly reduced α-SMA-positive MVECs (α-SMA[+] CD31[+] cells) in mitral leaflets compared to the control group (23.5 ± 3.5% versus 37.8 ± 1.9%; *p* < 0.05), which is indicative of effective EndoMT suppression (Figure 3C). Consequently, mitral leaflet area increased comparably (11%) in the LCZ696 group compared with the valsartan or control groups, albeit not statistically significant (Figure 3D). Neprilysin and natriuretic peptides were well expressed in in vitro hMVECs and B- and C- type natriuretic peptides’ expressions were significantly low in the diseased MVEC from the patient with functional MR (Figure 3E).

### 2.4. The Effects of Neprilysin Inhibitor in Human Mitral Valve Endothelial Cells In Vitro Study

**Figure 4:**
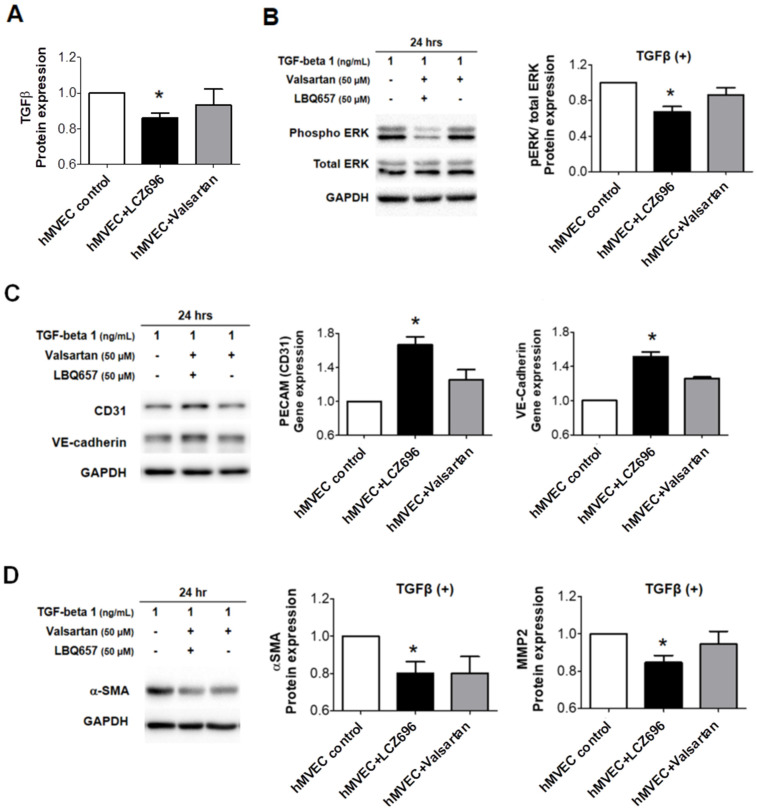
(**A**) TGF-β protein levels in human mitral valve endothelial cells (hMVECs) treated with LCZ696 or valsartan as determined by Western blots. Values represent the fold changes relative to the mean value measured in the control group. (**B**) Immunoblot analysis of total ERK, phosphorylated ERK, and GAPDH from hMVECs treated with LCZ696 or valsartan after TGF-β stimulation. Quantification of ERK density is relative to GAPDH. Values represent fold changes of the mean values relative to the control group. (**C**) Immunoblot analysis of CD31, PECAM, and GAPDH from hMVECs treated with LCZ696 or valsartan after TGF-β stimulation. Quantification of CD31 and PECAM density is relative to GAPDH. Values represent fold changes of the mean values relative to the control group. (**D**) Immunoblot analysis of α-SMA and GAPDH from hMVECs treated with LCZ696 or valsartan after TGF-β stimulation. Quantification of α-SMA density is relative to GAPDH. Values represent fold changes of the mean values relative to the control group. hMVECs indicates human mitral valve endothelial cells; TGF-β, transforming growth factor-β; MV, mitral valve; hrs, hours; ERK, extracellular signal-regulated kinase; GAPDH, glyceraldehyde-3-phosphate dehydrogenase; PECAM, platelet endothelial cell adhesion molecule; CD31, cluster of differentiation 31; VE-cadherin, vascular endothelial cadherin; α-SMA, α-smooth muscle actin; MMP, matrix metallopeptidase. * *p* < 0.05 for differences from the control group.

TGF-β protein expression was significantly lower in in vitro hMVECs treated with LCZ696, when compared to those in controls and hMVECs treated with valsartan (Figure 4A). LCZ696 attenuated downstream ERK phosphorylation after TGF-β stimulation (Figure 4B), increased CD31 and VE-cadherin expression (Figure 4C), and decreased α-SMA and MMP-2 expression significantly (Figure 4D). Our results suggest that LCZ696 can suppress EndoMT and thus mitigate inadequate leaflet adaptation, while valsartan does not have much effect on this process.

### 2.5. Neprilysin Inhibitor Attenuates Functional Mitral Regurgitation after Myocardial Infarction

**Figure 5:**
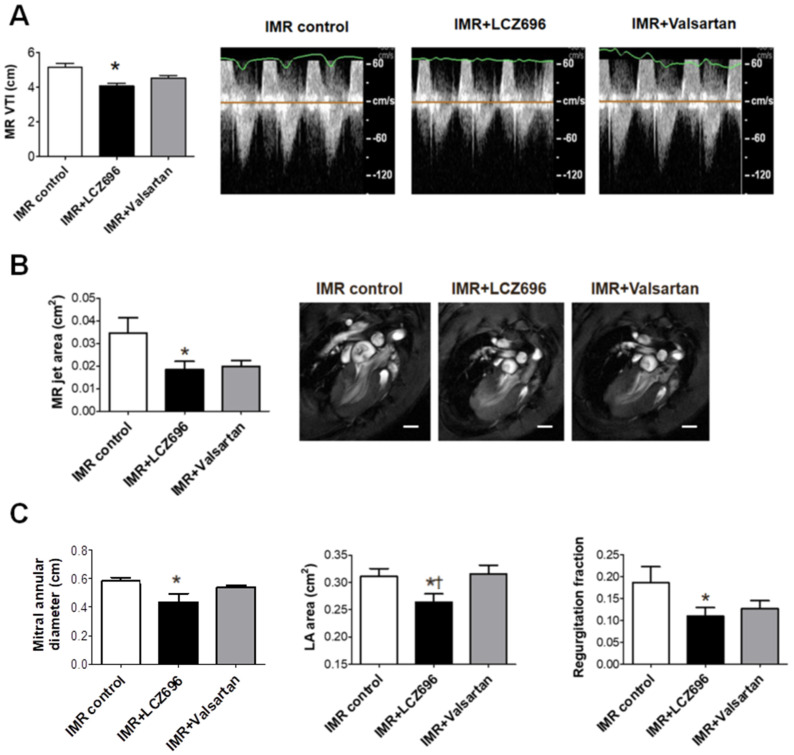
(**A**) Comparison of representative images from continuous-wave Doppler echocardiograms among the three groups at 6 weeks after the MI and a quantitative graph of MR VTI measurement. (**B**) Representative 3-chamber view images from cardiac MRI and a quantitative graph, which shows comparisons of MR jet area among the three groups. Scale bar = 3 mm. (**C**) Mitral annular diameter, left atrium (LA) area and regurgitant fraction were significantly lower in the LCZ696 group compared with the control group. The valsartan group was not significantly different from the control group. IMR indicates ischemic mitral regurgitation; VTI, time velocity integral; LA, left atrium. * *p* < 0.05 for differences from the control group. ^†^ *p* < 0.05 for differences from the valsartan group.

The extent of ischemic MR was evaluated using cardiac MRI and Doppler echocardiogram. The LCZ696 group showed significantly smaller MR VTI, which was acquired from continuous-wave (CW) Doppler echocardiogram than in the control group (Figure 5A) despite no significant differences in the change of arterial pressure between the treatment groups (Table 1). Cardiac MRI demonstrated that the MR jet area, mitral annular diameter and left atrium (LA) area were significantly lower in the LCZ696 group than in the control group (Figure 5B,C). Although the valsartan group showed numerical differences in MR jet area and regurgitant fraction compared with the control group, the differences were not statistically significant. Moreover, the LA area after 6 weeks of treatment was significantly larger in the valsartan group than in LCZ696 group (Figure 5C).

### 2.6. Functional Improvement in Pressure-Volume Loop Analysis and Treadmill Exercise Test

**Figure 6:**
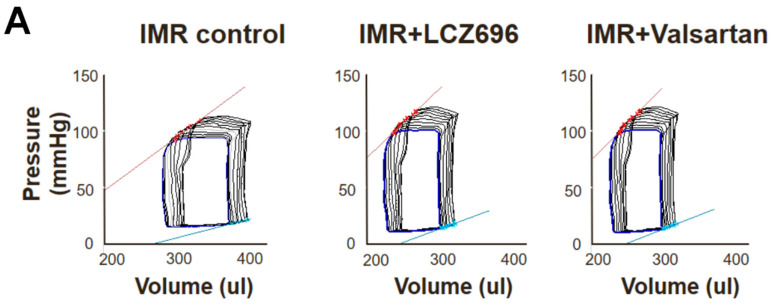

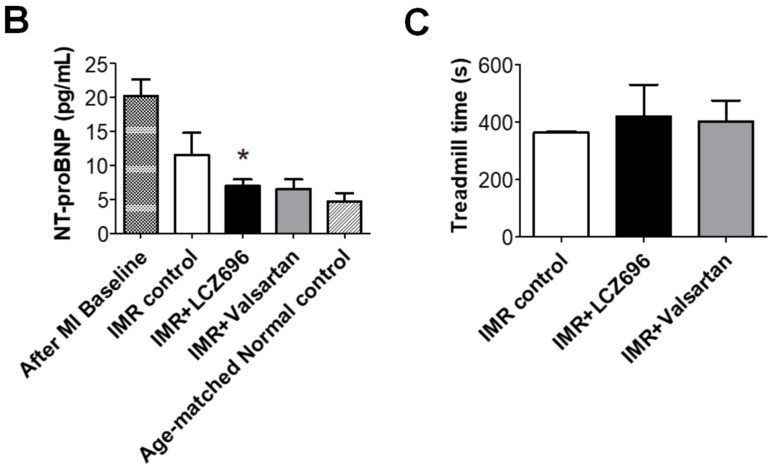
(**A**) Representative pressure-volume loops from the three groups. The LCZ696 and valsartan treatment groups had smaller left ventricular volume and higher end-systolic pressure-volume relation (red line) compared with the control group. (**B**) The NT-proBNP level after 6 weeks of LCZ696 treatment was significantly decreased compared with the baseline level immediately after MI and it was similar to that of the age-matched normal control group without MI. (**C**) Comparisons of treadmill exercise duration among the three groups at 6 weeks after MI. IMR indicates ischemic mitral regurgitation; NT-proBNP, N-terminal pro B -type natriuretic peptide; MI, myocardial infarction. * *p* < 0.05 for differences from the control group.

Although there were no significant differences in the change of LV ejection fraction (EF) or fractional shortening (FS) among the three groups when assessed by cardiac MRI or echocardiogram (Figure 2B,D), pressure-volume loop analysis showed that LCZ696 and valsartan treatment comparably increased ESPVR than the control group (Figure 6A) and dP/dt was significantly greater in the LCZ696 group than in the MR control group (8660 ± 388 mmHg/s versus 6940 ± 509 mmHg/s; *p* = 0.01) or in the valsartan group (5946 ± 853 mmHg/s; *p* = 0.02). The hemodynamic parameters are described in Table 1.

The LCZ696 group had significantly decreased NT-proBNP levels at 6 weeks after MI compared with the levels immediately after MI, whereas the control group did not. The NT-proBNP level after 6 weeks of treatment with LCZ696 was close to that of age-matched normal controls without MI (Figure 6B). The functional capacity of experimental animals was assessed by treadmill test after 6 weeks treatment and the LCZ696 group had higher exercise duration than the control group, albeit this difference was not statistically significant (Figure 6C).

## 3. Discussion

For patients with secondary functional MR, current medical treatment usually does not sufficiently reduce MR or reverse adverse LV remodeling [5]. Persistence of functional MR leads to high morbidity and mortality in patients managed with medical therapy [29]. 

Since ARNI, a novel complex of the ARB valsartan with a neprilysin inhibitor sacubitril, significantly improved clinical outcomes compared with an ACE inhibitor in the PARADIGM-HF trial [24], a treatment with ARNI is now recommended to replace ACE inhibitors or ARBs in symptomatic patients with HF and a reduced EF [30]. Importantly, in the recent PRIME trial [22], we found that ARNI treatment had a more favorable effect on reducing functional MR than ARB treatment alone. However, this trial was not an outcome study so these results cannot be used to determine whether ARNI can replace an ACE inhibitor or ARB in patients with secondary MR. Moreover, the way ARNI works for reduction of MR is not yet understood. In most ARNI trials including the PARADIGM-HF trial [24], the effect of ARNI on cardiac structure and function was not evaluated. As neprilysin inhibition has vasodilating effects and facilitates sodium excretion [25,26], combined inhibition of the renin-angiotensin system and neprilysin has greater hemodynamic and neurohormonal effects than ACE inhibition or ARBs alone and may affect LV remodeling profoundly [31,32]. However, the effects of ARNI on improvement of LV remodeling have not been examined.

The findings of the present study support the hypothesis that neprilysin inhibition facilitates LV reverse remodeling and suppresses EndoMT, which mitigates inadequate leaflet adaptation, resulting in reduction of functional MR after MI. This is the first animal study to evaluate the effect of ARNI on LV reverse remodeling using cardiac MRI and to focus on the direct molecular mechanism of inadequate adaptation of mitral leaflets. Our choice of cardiac MRI as an imaging tool for assessment of LV remodeling could be one particular strength of the present study because echocardiography is not as accurate as cardiac MRI for LV volume measurement in small animals such as rats, even though it is a standard imaging method for evaluation of MR. The PRIME trial did not identify significant differences of LV volume changes in part because we used echocardiography for evaluating LV volumes, and also because the follow-up duration and sample size may not have been sufficient to detect these changes [22]. 

In the present study, using an ischemic MR animal model, we found that in vivo LCZ696 treatment decreased myocardial fibrosis as well as LV volume and improved systolic function of the LV, which was assessed by invasive pressure-volume loop analysis. We also demonstrated that LCZ696 strongly attenuates fibrotic thickening of mitral leaflets and is also associated with a reduction in α-SMA and TGF-β expression. Significant decreases of α-SMA-positive MVECs in excised mitral leaflets after LCZ696 treatment may suggest that LCZ696 effectively suppresses EndoMT. Consequently, LCZ696 treatment seems to restore leaflet adaptation and adequate growth, while valsartan treatment alone does not have much effect on this process. Taking into consideration that an insufficient increase in mitral leaflet area in response to LV remodeling is associated with development of functional MR [18], our findings suggest an important mechanism of ARNI treatment in reducing functional MR. 

Furthermore, we evaluated primary cultured human MVECs to demonstrate the action mechanism of neprilysin inhibitor with a focus on molecular changes in vitro. Human MVECs were isolated from the human MV tissues in the hearts of recipients who received cardiac transplants and had functional MR. Neprilysin and natriuretic peptides were well expressed in hMVECs. Although the heterogeneity of MVECs obtained through primary culture could affect the results of the in vitro study, repetitive sets of experiments suggested that LCZ696 treatment significantly reduced TGF-β expression in MVECs, and attenuated downstream ERK phosphorylation. ERK mediates the TGF-β signaling which regulates various processes, including cell proliferation, differentiation and apoptosis. Abnormal TGF-β overexpression causes excessive EndoMT, which eliminates adaptive growth of tethered mitral leaflet.

Our study has several limitations. First, whereas the PRIME study was aimed at patients with chronic functional MR lasting over 6 months and who had a stable, optimized dose of HF medications for at least 4 weeks before screening, the present study was a 6-week animal experiment, which was not sufficient to assess chronic phase of MR. Extending the study period beyond this timeframe was not practical because the maximum allowable weight for the animals to enter the MRI machine is limited. However, our findings provide further evidence to support the substantial benefits of ARNI observed in a recent trial where initiation of ARNI therapy for acute decompensated HF led to a greater reduction in the NT-proBNP concentration than enalapril therapy [33]. Second, Doppler echocardiography does not accurately quantify MR extent in SD rats. Nevertheless, we could overcome this limitation with the use of cardiac MRI to evaluate MR jet areas, LA areas, mitral annular diameter and regurgitant fraction as well as LV volumes more accurately. Third, the ARB valsartan was chosen as an active control to assess the effect of neprilysin inhibitor treatment clearly even though ACE inhibitors remain the preferred choice for treatment of HF. We assumed ACE inhibitors and ARBs would have similar effects on functional MR because they have similar effects on LV remodeling [12]. It is also a limitation that the animal model was based on LV with preserved EF. Given that the functional MR is often associated with HF with reduced EF, this is a limit for translation of results in human setting.

In conclusion, neprilysin inhibitor treatment has positive effects on LV reverse remodeling and also directly modulates EndoMT in the mitral valve, which attenuates leaflet fibrosis and restores inadequate leaflet adaptation. Our results provide new insights into how treatment with ARNI may reduce secondary MR after MI.

## 4. Methods/Material

### 4.1. Experimental Animals

All animal experiments and protocols were performed in accordance with the Guide for the Care and Use of Laboratory Animals, and approved by the Institutional Animal Care and Use Committee of Asan Institute for Life Sciences (2018-14-191). In male SD rats weighing 250–350 g, functional MR was induced by occluding left circumflex coronary artery. Two weeks after MI, MR and LV dilatation were confirmed by echocardiography and cardiac MRI. Rats were randomly assigned to LCZ696 treatment (sacubitril/valsartan, 60 mg/kg/day in corn oil, *n* = 10), valsartan treatment (30 mg/kg/d in corn oil, *n* = 10), or corn oil only (MR control group; *n* = 11) group. Additional animals (*n* = 5) were also utilized to serve as age-matched sham controls. 

Until 6 weeks after MI, serial echocardiography and cardiac MRI were performed to quantify LV volumes, function, and extent of MR. At the end of the study, pressure-volume loop analysis was performed to assess the LV contractile function. The animals were then sacrificed with a bolus injection of potassium chloride (2 mEq) under anesthesia. Excised mitral leaflets and LV were fixed in 4% paraformaldehyde and analyzed by histopathology. Blood was drawn at baseline and at 6 weeks post-surgery. Serum was separated and stored at −80 °C until analysis.

### 4.2. Myocardial Infarction for Creating Ischemic, Functional Mitral Regurgitation

To create a model of ischemic, functional MR, MI was induced in male SD rats by occluding the left circumflex coronary artery, which shows color changes within lateral regions of LV myocardium. Briefly, rats were anesthetized with isoflurane (2~3%) and mechanically ventilated. A thoracotomy was performed at the level of 4th intercostal area. Hearts were then exposed through blunt dissection. The left circumflex coronary artery was tied off with silk suture (4-0). The chest was closed. Throughout the surgery, the body temperature was maintained by placing the animal under a heating lamp.

### 4.3. Cardiac Magnetic Resonance Imaging 

Rat cardiac MRI scans were performed using a 9.4 Tesla 160 mm system (Agilent, Santa Clara, CA, USA) and morphological cine images were acquired. Animals were anaesthetized with ~2% isoflurane and then placed in a cradle equipped with ECG leads, a respiratory sensor, and a heating pad. Long (2-chamber, 3-chamber, and 4-chamber views) and short-axis view images were acquired (TR, 260 ms; TE, 1.88 ms; cine frame, 20; effective cine TR, 11.9 ms; flip angle, 30°, average, 2; field of view, 50 × 50 mm; matrix, 192 × 192; slice number, 10 for short-axis, 1 for the remaining views; and slice thickness, 1.5 mm).

For each cardiac phase, end-diastolic volume (EDV) and end-systolic volume (ESV) from short-axis view images were computed by tracing the epicardial and endocardial borders. Short-axis slices from the apex to the base of the heart in end diastole and end systole were analyzed. The volumes were computed by summing the myocardial chamber areas corresponding to ventricular cavity and multiplying by the slice thickness (1.5 mm). In 3-chamber view images, MR flow was quantified by tracing the disturbed flow in the atrium that results from the regurgitant jet, which was signified by attenuated signal intensity. The atrial chamber size and mitral annular diameter were determined in 2- and 4-chamber view images in end systole.

### 4.4. Echocardiography

Doppler echocardiograms were obtained using an Affiniti 70C system equipped with a 12-MHz transducer (Philips Medical systems, Amsterdam, The Netherlands). Rats were anesthetized with 1–2% isoflurane. Parasternal long- and short-axis views were acquired, which was followed by M-mode imaging at the level of mid-papillary muscles. To assess mitral flow profile, color and continuous wave Doppler echocardiograms were performed. MR jets were recorded in apical 4-chamber view images and the time velocity integral (VTI) was measured off-line.

### 4.5. Pressure-Volume Loop Analysis

Rats were anesthetized (~2% isoflurane). A conductance catheter (Millar Instruments, Inc., Houston, TX, USA) was introduced via the right carotid artery and advanced into the left ventricular chamber. After 30 min of stabilization, baseline hemodynamics were recorded (MPVS Ultra; Millar Instruments). This was followed by a transient occlusion of the inferior vena cava to determine end-systolic pressure-volume relationship (ESPVR) and end-diastolic pressure-volume relationships (EDPVR). Hemodynamic parameters were analyzed off-line with LabChart Pro V8 (ADInstruments, Colorado Springs, CO, USA). The following data were determined: heart rate (HR), LV systolic pressure (LVSP), LV end-diastolic pressure (LVEDP), maximal/minimal rate of pressure development and decline (dP/dt max, dP/dt min) and ESPVR and EDPVR. 

### 4.6. Tissue Collection

At the end of pressure–volume loop analysis, animals received a bolus injection of potassium chloride (2 mEq) through the jugular vein. Hearts were excised and cannulated with a 16-gauge needle for retrograde perfusion via the aorta with 4% paraformaldehyde and fixed for 1 h. The hearts were stored at 4 °C until embedding. For tissue processing, the hearts were cut transversely, perpendicular to the left ventricular short-axis. The slices were embedded in paraffin and sectioned (3 µm) for immunohistochemical evaluation. 

### 4.7. Histology

Paraffin-embedded tissues were sectioned (3 µm). Hematoxylin/eosin (H&E) and picrosirius red (PSR) staining was performed according to the manufacturer specifications (Polysciences, 24901). The stained heart slices were photographed. The areas of fibrosis were digitized using ImageJ software (NIH, Bethesda, MD, USA). 

### 4.8. Immunohistochemical Staining

Tissues were sectioned and incubated with primary antibodies anti-TGFβ1 (Abcam, ab92486) and anti-α-smooth muscle actin (α-SMA) (Abcam, ab5694) at 4 °C overnight and washed. The sections were incubated with HRP-conjugated secondary antibody (Dako REAL™ EnVision™ Detection System, Peroxidase/DAB+ Rabbit/Mouse; K5007, DAKO) for 30 min at room temperature. The images were photographed by light microscopy and processed (ZEN pro).

### 4.9. Enzyme-Linked Immunosorbent Assay

Serum was separated (1500× *g*, −4 °C, 15 min) and stored at −80 °C until analysis. The N-terminal pro B-type natriuretic peptide (NT-proBNP) levels were determined using enzyme-linked immunosorbent assay (ELISA) kits according to the manufacturer specifications (CUSABIO, E08752R). 

### 4.10. Isolation of Human Mitral Valve Endothelial Cells and Cell Culture

Human mitral valve tissues were obtained from hearts of recipients who received cardiac transplants and had normal mitral valve leaflets and chords, but functional regurgitation. Mitral valve endothelial cells (MVECs) were isolated as previously described [34,35,36]. Briefly, human mitral valve tissues were digested in Dulbecco’s Modified Eagle’s Medium/F-12 (DMEM/F-12, Gibco 11330-032) containing collagenase I and II (1 mg/mL, each) with gentle agitation (37 °C) for 30 min. At the end of enzymatic digestion, the valve tissues were removed. The remaining media was centrifuged at 1200× *g* for 5 min. The pellet was cultured in DMEM/F-12 supplemented with 10% FBS (Gibco 16000-044), 1× antibiotic-antimycotic (Gibco 15240-062), and 0.1 mg/mL EC growth supplement (Corning 356006). MVECs were sorted by using fluorescence-activated cell sorting (FACS) and then only cluster of differentiation 31 (CD31), or platelet endothelial cell adhesion molecule (PECAM)-1 positive cells were cultured in a 1.5% gelatin coated dish.

### 4.11. In Vitro MVECs Study

MVECs were evaluated in vitro using a relatively early passage of the isolated cells. On the day of experiment, MVECs were serum-starved with DMEM/F-12 for 2 h and then switched to a regular DMEM/F-12 supplemented with endothelial cell growth factors, as described above. LBQ657, an active form of sacubitril (Cayman Chemical 19829) and valsartan, an angiotensin II receptor antagonist (Selleckchem S1894) were added at a final concentration of 50 μM, each. At the end of the study, the cells were lysed with RIPA buffer (Cell Signaling Technology 9806).

### 4.12. Western Blotting

MVECs were treated with RIPA buffer containing a cocktail of protease inhibitors (Roche, 11836153001). Proteins were extracted and quantified with a BCA protein assay kit (Thermo Scientific 23225). Samples (10 µg) were loaded onto 12% SDS-PAGE gels and transferred onto PVDF membranes. Blots were probed by anti-human vascular endothelial cadherin (VE-Cadherin) (1:1000, Cell Signaling Technology 2158S), CD31 (PECAM-1) (1:1000, Cell Signaling Technology 3528), matrix metallopeptidase-2 (MMP-2) (1:1000, Cell Signaling Technology 4022), α-SMA (1:1000, Abcam ab5694), extracellular signal-regulated kinase (ERK) (1:1000, Cell Signaling Technology 4695S), pERK (1:1000, Cell Signaling Technology 9101S), glyceraldehyde-3-phosphate dehydrogenase (GAPDH) (1:1000, Invitrogen MA5-15738) and visualized by chemiluminescence (ATTO), and quantified using ImageJ software.

### 4.13. Real-Time qPCR

MVEC gene expressions of endothelial and mesenchymal markers were determined by qPCR. RNA was extracted by RNeasy Mini Kit (QIAGEN). cDNA was synthesized using a cDNA synthesis kit according to the manufacturer specifications (Promega M1705). qPCR was performed in triplicate using an ABI 7500 system (Applied Biosystems, Waltham, MA, USA) each with 20 μL PCR reaction mix (17 μL SYBR-Green mix [Applied Biosystems 4367659], 2 μL forward/reverse primer [10 μM], and 1 μL cDNA sample). GAPDH was used as a housekeeping gene.

### 4.14. Statistical Analysis

Kruskal–Wallis tests were performed to determine differences among the groups. Unpaired t tests with Bonferroni correction were performed to assess mean differences between groups. The results were confirmed using Dunne’s multiple comparisons. If the unpaired t tests did not confirm Dunne’s test, then Dunne’s *p* value was reported (and noted with the Dunne’s test). All data are expressed as mean ± standard error of measurement (SEM).

## Figures and Tables

**Table 1:** Hemodynamic parameters 6 weeks after myocardial infarction.

Variables	IMR Control	IMR + LCZ696	IMR + Valsartan	*p* Value
SBP, mmHg	120.6 ± 3.0	118.7 ± 3.4	118.9 ± 4.0	0.925
DBP, mmHg	67.7 ± 3.9	66.2 ± 3.6	63.3 ± 3.0	0.559
MAP, mmHg	85.4 ± 2.8	83.7 ± 2.2	81.8 ± 2.8	0.483
HR, bpm	352 ± 23	336 ± 20	363 ± 16	0.152
LVESP, mmHg	106.6 ± 7.2	118.9 ± 4.4	102.7 ± 7.0	0.182
LVEDP, mmHg	7.7 ± 0.9	7.9 ± 1.0	11.2 ± 2.3	0.697
−dP/dt, mmHg/s	6940 ± 509	8660 ± 388 *^†^	5946 ± 853	0.019
+dP/dt, mmHg/s	7370 ± 504	8623 ± 273 ^†^	6799 ± 444	0.014
ESPVR, mmHg/µL	0.471 ± 0.22	0.621 ± 0.31	0.798 ± 0.50	0.142
EDPVR, mmHg/µL	0.126 ± 0.10	0.126 ± 0.07	0.279 ± 0.22	0.163

Values are presented as mean ± SEM. IMR indicates ischemic mitral regurgitation; SBP, systolic blood pressure; DBP, diastolic blood pressure; MAP, mean arterial pressure; HR, heart rate; LVESP, left ventricular end-systolic pressure; LVEDP, left ventricular end-diastolic pressure; -dP/dt min, minimal rate of pressure development and decline; +dP/dt, maximal rate of pressure development and decline; ESPVR, end-systolic pressure-volume relationship; EDPVR, end-diastolic pressure-volume relationship. * *p* < 0.05 for differences from the control group, ^†^ *p* < 0.05 for differences from the valsartan group.
